# Heparin Modulates the Endopeptidase Activity of *Leishmania mexicana* Cysteine Protease Cathepsin L-Like rCPB2.8

**DOI:** 10.1371/journal.pone.0080153

**Published:** 2013-11-21

**Authors:** Wagner A. S. Judice, Marcella A. Manfredi, Gerson P. Souza, Thiago M. Sansevero, Paulo C. Almeida, Cláudio S. Shida, Tarsis F. Gesteira, Luiz Juliano, Gareth D. Westrop, Sanya J. Sanderson, Graham H. Coombs, Ivarne L. S. Tersariol

**Affiliations:** 1 Centro Interdisciplinar de Investigação Bioquímica, Universidade de Mogi das Cruzes, Mogi das Cruzes, Brazil; 2 Departamentos de Bioquímica, Universidade Federal de São Paulo, São Paulo, Brazil; 3 Instituto de Ciência e Tecnologia, Universidade Federal de São Paulo, São José dos Campos, Brazil; 4 Division of Developmental Biology, Cincinnati Children's Hospital Medical Center, Cincinnati, Ohio, United States of America; 5 Departamentos de Biofísica, Universidade Federal de São Paulo, São Paulo, Brazil; 6 Strathclyde Institute of Pharmacy and Biomedical Sciences, University of Strathclyde, Glasgow, Scotland; Johns Hopkins Bloomberg School of Public Health, United States of America

## Abstract

**Background:**

Cysteine protease B is considered crucial for the survival and infectivity of the *Leishmania* in its human host. Several microorganism pathogens bind to the heparin-like glycosaminoglycans chains of proteoglycans at host-cell surface to promote their attachment and internalization. Here, we have investigated the influence of heparin upon *Leishmania mexicana* cysteine protease rCPB2.8 activity.

**Methodology/Principal Findings:**

The data analysis revealed that the presence of heparin affects all steps of the enzyme reaction: (i) it decreases 3.5-fold the *k*_1_ and 4.0-fold the *k*_−1_, (ii) it affects the acyl-enzyme accumulation with pronounced decrease in *k*_2_ (2.7-fold), and also decrease in *k*_3_ (3.5-fold). The large values of Δ*G  = * 12 kJ/mol for the association and dissociation steps indicate substantial structural strains linked to the formation/dissociation of the ES complex in the presence of heparin, which underscore a conformational change that prevents the diffusion of substrate in the rCPB2.8 active site. Binding to heparin also significantly decreases the α-helix content of the rCPB2.8 and perturbs the intrinsic fluorescence emission of the enzyme. The data strongly suggest that heparin is altering the ionization of catalytic (Cys^25^)-S^−^/(His^163^)-Im^+^ H ion pair of the rCPB2.8. Moreover, the interaction of heparin with the *N*-terminal pro-region of rCPB2.8 significantly decreased its inhibitory activity against the mature enzyme.

**Conclusions/Significance:**

Taken together, depending on their concentration, heparin-like glycosaminoglycans can either stimulate or antagonize the activity of cysteine protease B enzymes during parasite infection, suggesting that this glycoconjugate can anchor parasite cysteine protease at host cell surface.

## Introduction

Papain-like cysteine proteases have been identified in parasitic organisms, such as *T. cruzi* (cruzain), *T. brucei* (trypanopain, TbCatB) and different *Leishmania* spp. (CPA, CPB, CPC) [1]. The cysteine proteinase (CP) activity of *Leishmania mexicana* is considerably greater in the mammalian amastigote form than in the promastigote forms [2]. These classes of CPs exist as multiple isoenzymes [3]–[17], which are encoded by a tandem array of 19 similar CPB genes [7]–[11]. The CPs, together with homologues from either leishmanias [12], [13] and trypanosomatids such as *Trypanosoma cruzi* (cruzipain or cruzain) [14], [15] and *Trypanosoma brucei* [16] are cathepsin L-like and are characterized by the presence of an unusual 100 amino acid *C*-terminal extension, which in some cases is highly glycosylated. Like cruzain, CPB from *L. mexicana* are abundant and stage-regulated and can occur on the surface of parasite [10]–[13].

CPB are thought to be crucial for the survival and infectivity of the parasite in its human host and have been involved in successful invasion of host macrophages by promastigotes, the transformation of parasitic forms, parasitic nutrition, and evasion of hosts immune system [10], [11], [17]–[19]. Because of the importance of cysteine proteases in the survival and in the life cycle of *Leishmania*, they have been targets for development of inhibitors as antileishmanial drugs [10], [20], [21].

A recombinant form of the enzyme encoded by CPB2.8 but lacking the *C*-terminal extension (known as CPB2.8ΔCTE) was expressed [22], and its substrate specificity has been studied extensively [23]–[26], [27] and several peptide inhibitors have also been reported for it [28]–[30]. The rCPB2.8 presents the amino acids Asn^60^, Asp^61^, Asp^64^ in the α-helices that form the wall of the active site cleft [27], [31].

Heparan sulfate proteoglycans are ubiquitous components of cell surface of animal cells. They are components of plasma membranes and also the extracellular matrix (ECM). Heparan sulfate is sulfated glycosaminoglycans composed of disaccharides containing uronic acid and glucosamine with *N*- and 6-*O*-sulfates and *N*-acetyl substitutions. These molecules are located to regulate the cell interactions with the environment. The interaction of heparan sulfate or heparin-like glycosaminoglycans with proteins control a large spectrum of biological processes including cell homeostasis, growth factor activity, cell adhesion and parasitic infection [32], [33].

Several microorganism pathogens bind to the glycosaminoglycan chains of proteoglycans at the host cell surface to promote their attachment and internalization [34]. Heparan sulfate proteoglycan-binding mediates the interaction of the amastigote form of *Leishmania amazonensis* to mammalian cells, this interaction seems to be an important first step in the host cell invasion by *Leishmania* [35]. Recently, it has been suggest that a cell surface metalloproteinase from promastigote form of *L.*(*V.*) *braziliensis* can interact with heparin-like glycosaminoglycans of vector Lulo cells [36]. Interesting, secreted cysteine protease (CPB) can participate in *Leishmania* infection by degradation of fibronectin of the host’s extracellular matrix (ECM), thereby facilitating the local spread of the parasite [37].

We have shown that glycosaminoglycans (GAGs), especially heparin-like compounds, can modulate the catalytic activity some papain-like enzymes [38], [39]. Heparan sulfate and heparin are able to interact with cathepsin B specifically; this interaction promotes the stabilization of the enzyme in neutral/alkaline pH, favoring the endopeptidasic activity of cathepsin B at neutral pH [39]. Also, the release of kinin by *T. cruzi* trypomastigotes was increased 10-fold in the presence of heparan sulfate. Previous data showed that heparan sulfate markedly potentiates the kininogenasic activity of cruzipain by forming a heparan sulfate-kininogen-cruzipain ternary complex [40]. Also, it has been shown that chondroitin sulfate proteoglycans are able to activate the collagenolytic activity of cathepsin K [41].

Therefore, the interaction of *Leishmania* cysteine protease with GAGs of the host cell surface may be of significant interest for understanding the biological role of this class of enzyme in degradation of host ECM components in parasite infection. This study addressed this possibility.

## Materials and Methods

### Enzyme rCPB2.8

The recombinant CPB2.8ΔCTE (designated rCPB2.8 throughout this manuscript is a recombinant cysteine protease type B truncated in the C-terminal) was obtained and purified as earlier described [22], [25]. The rCPB2.8 expressed in *E. coli* show 38000-*M*_r_ and it is converted to 26000-*M*_r_ activity mature form after incubation in acidic medium at 37 °C in the presence of 100 mM sodium acetate buffer, pH 5.5, 0.9 M NaCl, 2 mM EDTA (Ethylenediaminetetraacetic acid), and 10 mM DTT (Dithiothreitol) until full conversion was observed (approx. 2 — 4 h). *N*-terminal Pro-region peptides released during the activation process are removed by gel filtration at room temperature using a 50 mL Sephadex G-50 column (Amersham Pharmacia Biotech) equilibrated in 100 mM sodium acetate, pH 5.5, 2 mM EDTA, 10 mM DTT, 0.45 M NaCl, and 0.01% (v/v) Triton X-100 at a flow rate of 0.75 mL/min over 55 mL.

The enzyme concentration was determined by the titration of the active site using the irreversible inhibitor E64 (1-[[(L-trans-epoxysuccinyl)∼L∼leucyl]amino]-4-guanidino-butane). Aliquots of the rCPB2.8 were incubated in the presence of different concentrations of E64 in 100 mM sodium acetate buffer, 5 mM DTT, 20% glycerol (included in the buffer assay to increase the enzyme stability), pH 5.5 at 35°C for 10 min. The uninhibited remaining enzyme was monitored by the hydrolysis of Z-FR-MCA (Carbobenzoxyl-L-phenylalanil-L-arginine-4-methylcoumarinyl-7-amide) in the wavelengths set at 360 nm for excitation and 480 nm for emission on a thermostatic Hitachi F-2500 spectrofluorometer.

### Cloning, expression and purification of the N-terminal pro-region of *Leishmania mexicana* CPB2.8

The entire *N*-terminal pro-region of CPB2.8 was generated using PCR (Polymerase Chain Reaction) and the plasmid containing the N-terminally His_6_ tagged rCPB2.8 sequence as template [22]. The primers used were 5′GCCATATGACGCCGGCTGCTGCGCTGTTCG3′ and 5′TAACTCGAGCGACAGGTCTGC GCGCGCCTTG3′. The PCR product was cloned into pET21a+ (Novagen) with *Nde*I and *Xho*I to yield a construct to express the *N*-terminal pro-region with a C-terminal His_6_ tag. The vector was transformed into *Escherichia coli* BL21DE3 for expression overnight at 37°C with expression being induced with 0.5 mM isopropyl β-D-thiogalactoside. The protein was purified from inclusion bodies and soluble phase using nickel agarose chromatography under denaturing conditions as described in [22]. Following dialysis of the sample from 8 M urea to PBS *(*Phosphate Buffered Saline) pH 7.4 the majority of the protein sample remained in solution and was used for analysis. The purified protein gave a single major band on SDS/PAGE (Sodium Dodecyl Sulphate/Poliacrylamide gel electrophoresis).

### Enzyme assays

The influence of heparin upon rCPB2.8 endopeptidase activity was monitored fluorometrically using the fluorogenic substrate Z-FR-MCA. The fluorescence intensity was monitored on a thermostatic Hitachi F-2500 spectrofluorometer. The steady-state kinetic assays with fluorogenic substrate were performed in 100 mM sodium acetate buffer (pH 5.5) containing 20% glycerol and 5 mM DTT at 35°C. The enzyme was activated by its pre-incubation in the assay buffer for 5 min at 35°C before the substrate addition. The concentration of active rCPB2.8 was determined by titration with its irreversible inhibitor E-64. All reactions were done in 1×1 cm cross section quartz cuvette. For the Z-FR-MCA (0.1 — 10 µM) substrate assays, the excitation and emission wavelengths were set at 360 and 480 nm, respectively. The kinetic parameters were determined by measuring the initial rate of hydrolysis at various substrate concentrations in the absence or in the presence of heparin (0 — 60 µM). In the experiments we have used a size-defined (10 kDa) bovine lung heparin (The Upjohn Co), prepared by using size exclusion column approach [32]. The fluorescence of 7-amino-4-methylcoumarin and ortho-aminobenzoic acid were determined for the calculation of precise rate constants. All kinetic experiments were performed in triplicate. In order to calculate the concentration of the released product calibration curves of fluorescence versus concentration were constructed. The data obtained were analyzed by nonlinear regression using the program GraFit 5.0 (Erithacus Software Ltd.). The data were analyzed in steady-state kinetic system and the values for the constants were determined by using nonlinear regression to non-competitive Equation 1.
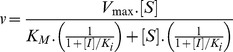
(1)


The influence of others high sulfated GAGs namely heparan sulfate, chondroitin sulfate, and dematan sulfate were analyzed by determination of inhibitory potential IC_50_. The enzyme rCPB2.8 activity was performed in 100 mM sodium acetate buffer (pH 5.5) containing 20% glycerol and 5 mM DTT at 35°C pre-activated for 5min. The substrate Z-FR-MCA hydrolyses was followed in a specrofluorometer F2500 in the excitation and emission wavelengths set at 360 and 480 nm, respectively, in the absence and in the presence of different concentrations of GAGs. The residual enzyme activity was registered and the rate values were plotted against GAGs concentrations and the data analyzed by nonlinear regression using the program GraFit 5.0 (Erithacus Software Ltd.) using the equation 2. The GAG effects on the enzyme activity were monitored at different concentrations (0 — 60 µM). All kinetic experiments were performed in triplicate.
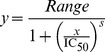
(2)


where *Range* is fitted uninhibited value and *s* is a slope factor. The equation assumes that *y* falls with increasing *x*.

### Determination of Individual Rate Constants for the Hydrolysis of Z-FR-MCA by rCPB2.8

Although the three-step mechanism for cysteine protease-catalyzed hydrolysis of peptides is widely accepted [59]–[61] **,** the individual rate constants that describe the steps have not been determined in the studies of rCPB2.8 inhibition. Since *k_cat_ = (k_2_.k_3_)/(k_2_+k_3_)*; *K_S_ = (k*_−*1*_ *+k_2_)/k_1_* and *K_M_ = K_S_.k_3_/(k_2_+k_3_)* are composite parameters of the rate constants, the Michaelis-Menten kinetic parameters *k_cat_* and *K_M_*, determined by steady-state analysis do not give a detailed picture about the kinetic mechanism of a cysteine protease. Clearly, the only way to get an accurate picture of rCPB2.8 inhibition by heparin is through the determination of the heparin effect upon the mechanistic kinetic parameters *k_1_*, *k*_−*1*_, *k_2_* and *k_3_*.

Based on the temperature dependence upon the kinetics parameters *k_cat_/K*_M_ and *k_cat_* for hydrolysis of Z-FR-MCA by the rCPB2.8 enzyme, we evaluated the individual constants *k*_1_, *k*_−1_, *k*_2_ and *k*_3_ of the hydrolytic reactions in the absence or in the presence of 40 µM heparin using the procedure reported by Ayala and Di Cera [42] and Judice *et al*. [43], [44] which was based on the assumption that the hydrolytic process of a cysteine protease occurs as described below:

The values of the kinetic parameters *k_cat_* and *K*_M_ for rCPB2.8 was determined using 100 mM sodium acetate buffer, 20% glycerol, 5 mM DTT, pH 5.5 activating the enzyme for 10 min in the temperature range 10°C to 35°C. In the temperature above of 35°C the only modifications were: 1) each reaction at temperatures higher than 35°C up to 55°C were started by addition to the reaction mixture of enzyme previously activated at a lower temperature using 5 mM DTT, temperature and the initial velocity was registered in the first 60 sec; 2) the buffer pH was corrected for the temperature used.

**Figure 1:**
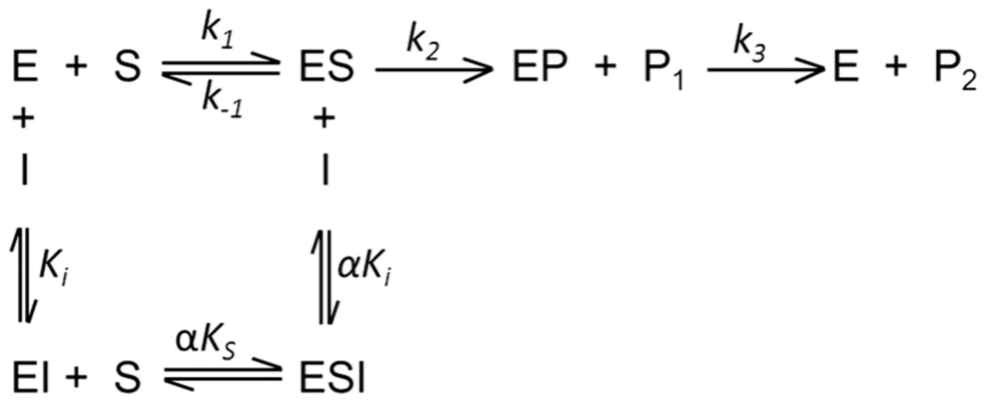
Schematic mechanism of hydrolytic process of a cysteine protease and inhibition. Individual constants *k*_1_, *k*_−1_, *k*_2_ and *k*_3_ of the hydrolytic reactions of cysteine protease. *k_1_* is the substrate diffusion constant into the active site, *k_–1_* the substrate dissociation constant, *k_2_* the acylation constant, *k_3_* the deacylation constant, *K_S_  =  (k*_−*1*_ *+k_2_)/k_1_*, *K*_i_ the inhibition constant, and α is the parameter of *K*_S_ and *K*_i_ perturbation.



The following equations were developed as earlier described [42]–[44]. The temperature dependence of rate constants obeys the Arrhenius law (Equation 3):
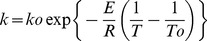
(3)


where *E* is the activation energy associated with the rate constant *k*, *R* is the gas constant, *T* the absolute temperature and *k_o_* the value of *k* at the temperature *T_o_* = 298,15 K. The equations that define the Michaelis-Menten parameters *k_cat_ /K*_M_ and *k_cat_*, which are based on the reaction of Figure 1, are as follows:

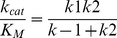
(4)

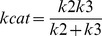
(5)

The substitution of Equation 3 in the Eq. 4 and Eq. 5 results in the following expressions: 
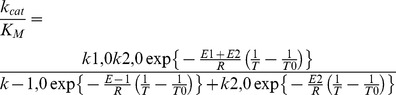
(6)

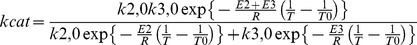
(7)


in which *k*_1,0_, *k*_−1,0_, *k*_2,0_ and *k*_3,0_ are the rate constants *k*_1_ (association constant)_,_ *k*_−1_ (dissociation constant), *k*_2_ (acylation constant) and *k*_3_ (deacylation constant), respectively at the temperature T_0_  =  298,15 K and *E_1_, E*_−*1*_ *, E_2_* and *E_3_* are the corresponding activation energies.

From the plot of ln *s* versus 1/T and ln *k_cat_* versus 1/T, as indicated in the equations above (Equations 5 and 6), the parameters *k_1,0_*, *k_–1,0_*, *k_2,0_*, *k_3,0_*, *E_1_, E*_−*1*_ *, E_2_* and *E_3_* can be determined for the temperature T_0_  =  298,15 K.

The Eyring transition-state theory allows the calculation of the entropy and enthalpy of activation using the following equation [43]–[47]:
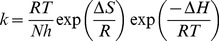
(8)


The substitution of Equation 8 in the Eq. 4 and Eq. 5 and calculating *k*_cat_/*K*_M_.T and *k_cat_* /T results in the following expressions: 
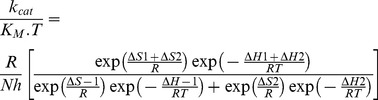
(9)

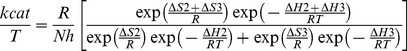
(10)


in which *N* is Avogrado’s number and *h* is Planck’s constant and *ΔS_1_, ΔS*_−*1*_ *, ΔS_2_* and *ΔS_3_* are corresponding activation entropies and *ΔH_1_, ΔH*_−*1*_ *, ΔH_2_* and *ΔH_3_* are corresponding activation enthalpies. *ΔS_1_, ΔS*_−*1*_ *, ΔS_2_, ΔS_3_*, *ΔH_1_, ΔH*_−*1*_ *, ΔH_2_* and *ΔH_3_* can be obtained from parameters using the Eyring plot ln(*k*_cat_ /*K*_M_.T) versus 1/T and ln(*k_cat_* /T) versus 1/T.

The values of the kinetic parameters *k_cat_* and *K*_M_ were determined for rCPB2.8 in the temperature range from 10°C to 55°C were: 1) each reaction at temperatures higher than 35°C was started by addition to the reaction mixture of enzyme previously activated at a lower temperature using 5 mM DTT, and the initial velocity was registered in the first 60 sec; 2) the buffer pH was corrected for the temperature used.

### rCPB2.8 pH Activity Profile

For the determination of pH activity profiles, the kinetics of Z-FR-MCA substrate hydrolysis were performed in absence or in presence of 40 µM heparin at 35 °C in four-component buffer system of constant ionic strength, consisting of 25 mM glycine, 25 mM acetic acid, 25 mM Mes and 75 mM Tris, containing 20% glycerol and 5 mM DTT, the pH of buffers were adjusted using HCl or NaOH diluted solutions. The substrate concentrations were kept 20-fold above the *K*_M_ values. The progress of the reaction was continuously monitored by the fluorescence of the released product and the initial rates were determined. It is important to mention that the enzyme was stable at pH range studied, and the pH values did not affect the ionic form of substrate. The pH activity profiles data were fitted according to Equation 11 by using non-linear regression software system (GraFit version 5.0, Erithacus Software Ltd) as follows: 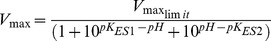
(11)


where 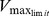
stands for the pH-independent maximum reaction rate, and p*K*_ES1_ and p*K*_ES2_ are the dissociation constants of a catalytically competent base and acid in the presence of the substrate respectively.

### Effect of Heparin upon Intrinsic Fluorescence of the rCPB2.8

The intrinsic fluorescence of tryptophan residues of the rCPB2.8 was monitored in 50 mM sodium phosphate buffer (pH 7) containing 20% glycerol at 35°C by measuring the emission of fluorescence between at 300 — 450 nm (10 nm slit) after excitation at λ_ex_  =  290 nm (5 nm slit) in a Hitachi F-2500 spectrofluorimeter in the absence or in the presence of different concentrations of heparin (0 — 167 µM). The 1 cm path-length cuvette containing 1 mL of the buffered enzyme solution (1 µM) was placed in a thermostatically controlled cell compartment under constant magnetic stirring in the spectrofluorimeter for 5 min prior to the addition of small aliquots of a highly concentrated heparin solution with minimal dilution (less than 5%) and the decrease in fluorescence signal was read. The dependence of the relative fluorescence change, i.e., Δ*F*  =  (*F*_obs_ – *F*_0_) where F_0_ is the initial rCPB2.8 solution fluorescence value and *F*_obs_ is the observed fluorescence value after each addition of heparin, was analyzed by nonlinear least-squares data fitting by the binding Equation 11 according to [48], [49], using Grafit 5.0 Software. The same procedure described above was used to measure the effect of heparin upon the intrinsic fluorescence of the *N*-terminal pro-region domain of CPB2.8.
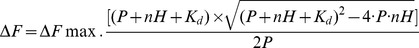
(12)


where *P* is the total protein concentration, *H* represents the added heparin fragments concentration, *n* the stoichiometry, *K*_d_ the dissociation constant and Δ*F*_max_ is the maximum fluorescence change.

### Circular Dichroism Experiments

Circular dichroism (CD) measurements in far ultraviolet regions (190 — 260 nm) of rCPB2.8-heparin interactions were conducted in a JASCO J-810 spectropolarimeter scanning at rate of 50 nm/min at 35 °C. Cells of 0.1 cm for the far UV were used. The experiments were done in 5 mM sodium phosphate buffer pH 5.8 containing 2 µM of enzyme rCPB2.8. The observed ellipticity was normalized to units of degrees.cm^2^.dmol^−1^. All dichroic spectra were corrected by subtraction of the background for the spectrum obtained with buffer alone or buffer containing heparin fragments. The CD spectra for the rCPB2.8 was analyzed for the relative amount in percentage, of the secondary structural elements by a program based on comparison to the spectra obtained for the structures of known proteins according to [50].

### Effects of heparin on the inhibitory activity of N-terminal pro-region of the enzyme rCPB2.8

The inhibitory effect of the *N*-terminal pro-region on the endopeptidase activity of the enzyme rCPB2.8 was determined in the absence or in the presence of 40 µM heparin. The enzyme rCPB2.8 was pre-activated by incubation in 50 mM sodium acetate buffer (pH 5.5), containing 5 mM DTT, 20% glycerol for 10 min at 35°C. The rCPB2.8 aliquots were incubated at different concentrations of pro-region (0 — 0.24 µM) and or with *N*-terminal pro-region previously pre-incubated for 30 min with heparin, the final concentration of heparin at enzyme assay was 12 µM. The endopeptidase activity of the enzyme was continuously monitored by the hydrolysis of fluorogenic substrate Z-FR-MCA in a spectrofluorometer F-2500 Hitachi, set at wavelengths 360 nm for excitation and 480 nm for emission. The remaining activities of the enzyme inhibited by *N*-terminal pro-region in the absence or in the presence of heparin were analyzed by nonlinear regression using the software GraFit 5.0 (Erithacus Software Ltda).

## Results

### Effect of Heparin upon the rCPB2.8 Endopeptidase Activity

The effect of heparin upon the rCPB2.8 endopeptidase activity was monitored with the aid of its fluorogenic substrate Z-FR-MCA, covering the rCPB2.8 subsites from S_2_ to S′_1_ [51], [52]. The HPLC and Maldi-TOF mass spectrometry analysis showed that Arg-MCA is the only peptide bond cleaved by rCPB2.8 on this substrate; also, heparin did not change the pattern of cleavage of this substrate by rCPB2.8. No interaction between Z-FR-MCA substrate with heparin was detected by studying the molar absorption of substrate in function of heparin concentration (data not shown). The possible effect of other high sulfated GAGs, namely heparan sulfate, chondroitin sulfate, and dermatan sulfate were also investigated upon rCPB2.8-catalysed hydrolysis of the fluorogenic substrate Z-FR-MCA. The results showed that the chondroitin sulfate, and dermatan sulfate are not able to inhibit the activity of rCPB2.8 in the concentration range from 0 – 60 µM (data not shown), and only heparin and heparan sulfate were capable of inhibit the endopeptidase activity of rCPB2.8. These results show that the interaction of heparin and heparan sulfate polysaccharides to rCPB2.8 is specific to heparin-like GAGs similar results have been observed previously [38], [39].

**Figure 2:**
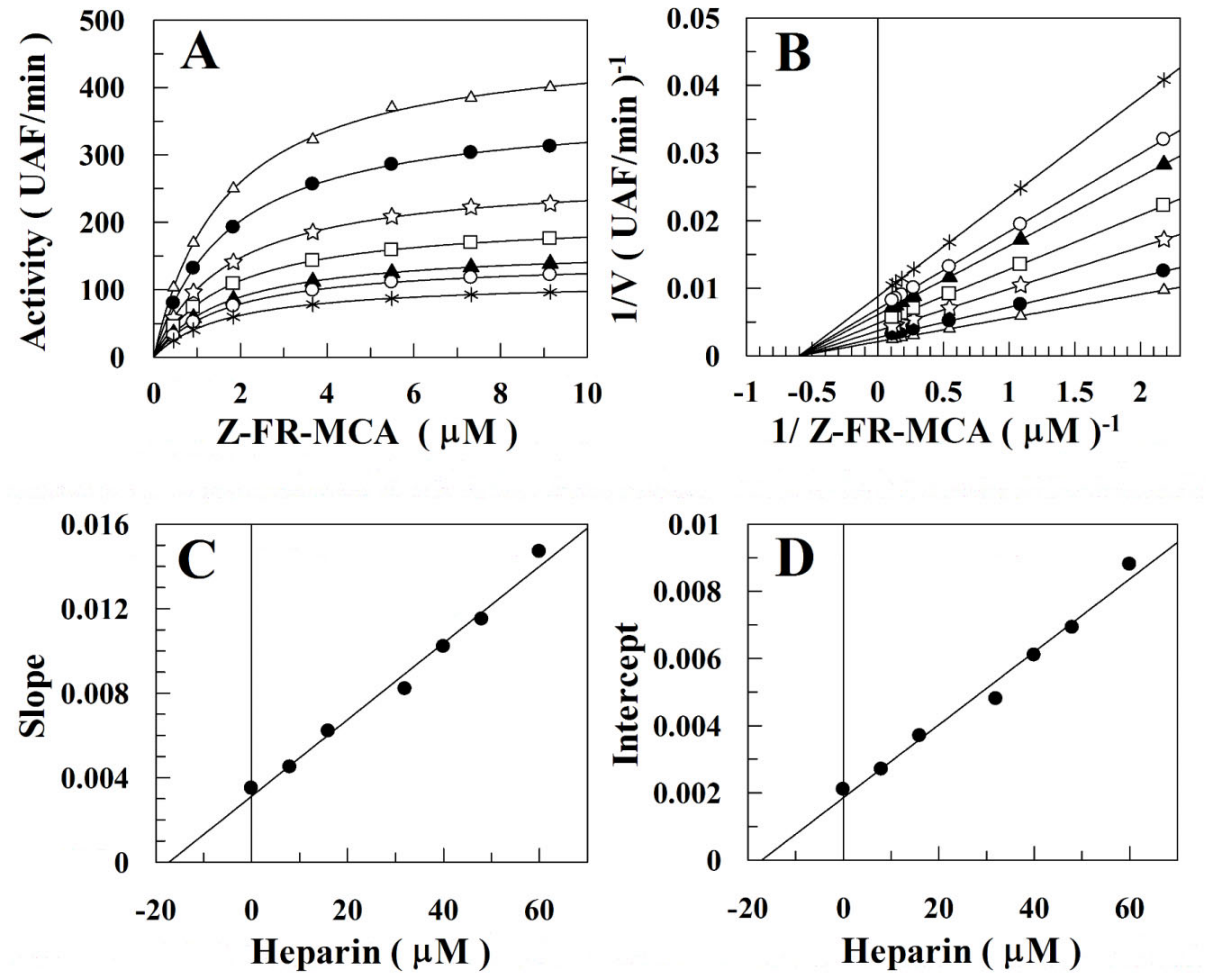
Effect of Heparin upon the rCPB2.8 Endopeptidase Activity. The influence of heparin upon rCPB2.8 endopeptidase activity was monitored fluorometrically using the fluorogenic substrate Z-FR-MCA. The steady-state kinetic assays with fluorogenic substrate were performed in 100 mM sodium acetate buffer (pH 5.5) containing 20% glycerol and 5 mM DTT at 35°C. The enzyme was activated by its pre-incubation in the assay buffer for 5 min at 35°C before the substrate addition. *A*, the rate of substrate Z-FR-MCA hydrolysis as a function of substrate concentration. The kinetic parameters were determined by measuring the initial rate of hydrolysis at various substrate concentrations in the absence or in the presence of heparin, control (▵–▵); 8 µM (•–•); 16 µM (•–•); 32 µM (□–□); 42 µM (▴–▴); 48 µM (○–○); 62 µM (*–*) of heparin. *B* **,** the reciprocal plot 1/*V* versus 1/[S] in the presence of different concentrations of heparin (0 — 62 µM). *C*, replots of *slope*_1/[S]_ versus [heparin]. *D*, replot of 1/*V*_−axis_ intercept versus [heparin]. The data of replots were taken from the reciprocal plot 1/*V* versus 1/[S].

The interaction of heparin with rCPB2.8 perturbs its catalytic activity upon Z-FR-MCA substrate (Fig. 2). The efficiency of the system for the hydrolysis of Z-FR-MCA can be altered by changing either *K*_M_ value (α parameter) or *k*_cat_ value (β parameter). Figs 2A and 2B show that the presence of heparin results in a large decrease in *V*_max_ value without changing the apparent binding affinity of the enzyme for its substrate Z-FR-MCA (*K*_S_  =  1.6±0.1 µM). It was observed that heparin binds to free rCPB2.8 (E) and to the enzyme-substrate complex (ES) with the same dissociation constant *K*_H_  =  17±1 µM (Figs 2D and 2E). The data analysis show that heparin inhibited rCPB2.8 by a linear non-competitive inhibition mechanism, α  =  1.0±0.1 and β  =  0 as depicted in Figure 1, where the ternary complex enzyme-substrate-inhibitor (ESI) cannot form product and can only be converted back to the ES complex or the EI complex.

Interesting, heparin showed a very similar kinetic pattern of inhibition upon papain when this enzyme was also assayed with the same substrate Z-FR-MCA. Heparin promoted a large decrease of 5.5-fold in Z-FR-MCA hydrolysis second-order rate without changing the affinity of papain for the Z-FR-MCA, showing a partial non-competitive inhibition behavior [38]. On the other hand, it has been demonstrated that the inhibitory effects of heparin on other cysteine proteases from the papain family may be related to the substrate structure [44], [53].

Seminal works of Schneck et al., 2008, and Steiner et al., 1987, have shown that the steady-state kinetic parameters *k_cat_*, *K_M_*, and *k_cat_/K_M_* do not give a clear picture of cysteine-proteases [54] and serine-proteases [55] substrate specificity, because *k_cat_*, *K_S_*, and *K_M_* are composite parameters of the intrinsic rate constants. Therefore, the determination of the steady-state parameters is not sufficient to describe the kinetic mechanism in the case of rCPB2.8 inhibition by heparin. In order to get an accurate picture of rCPB2.8 inhibition by heparin, we also analyzed the effect of heparin upon the individual rate constants *k*_1_, *k*_−1_, *k*_2_ and *k*_3_ for the substrate hydrolysis.

### Determination of the Individual Rate Constants in the rCPB2.8 Kinetic Mechanism

We have used temperature studies of kinetic parameters to resolve the individual rate constants *k_1_*, *k*_−*1*_, *k_2_* and *k_3_* in the kinetic mechanism of rCPB2.8 in the absence or in the presence of 40 µM heparin as previously described [42]–[47]. Measurements of *k*_cat_ */K*_M_ and *k*_cat_ as a function of temperature can resolve all the parameters depicted in Equations 5, 6, 8 and 9.

**Figure 3:**
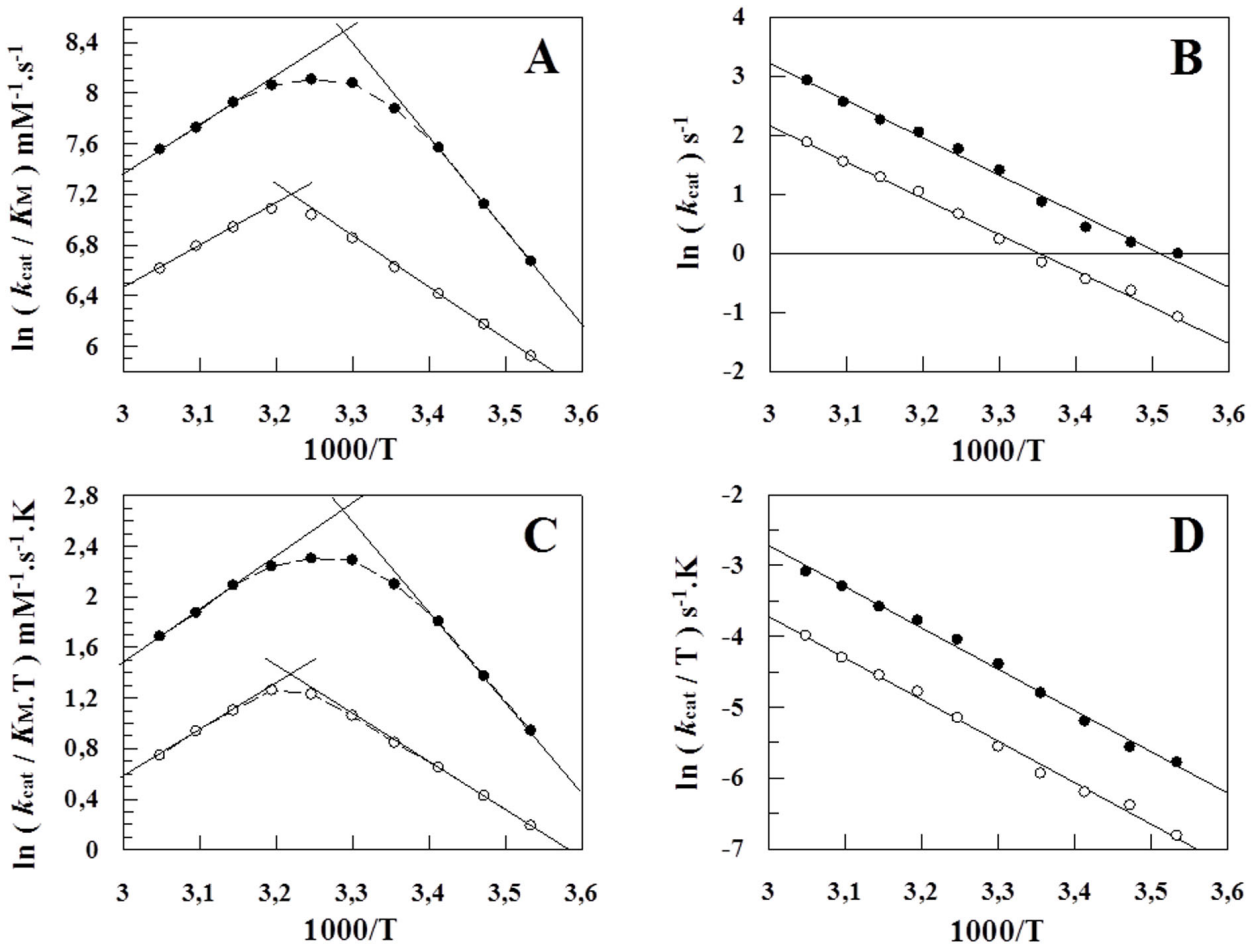
Arrhenius and Eyring plots of the *k_cat_/K_M_* and *k_cat_* for the Z-FR-MCA hydrolisys by rCPB2.8. *A*, Arrhenius plot of the *ln* (*k_cat_/K*_M_ *)* versus 1000/T. *B*, Arrhenius plot of the *ln k*_cat_ versus 1000/T. *C*, Eyring plot of the *ln (k_cat_/K*_M_.T*)* versus 1000/T. *D*, Eyring plot of the *ln (k_cat_/T)* versus 1000/T. The experimental conditions were: the values of the kinetic parameters *k_cat_* and *K*_M_ for rCPB2.8 was determined using 100 mM sodium acetate buffer, 20% glycerol, 5 mM DTT, pH 5.5 activating the enzyme for 10 min in the temperature range 10°C to 55°C as described under “Methods.” The data were obtained in the absence (•–•) or in the presence (○–○) of 40 µM heparin. The continuous lines (*slops*) were drawn according the Equations 6, 7, 9 and 10, with the best-fit parameter values listed in Table 1 and 2.

The temperature dependence of *k*_cat_ */K*_M_ and *k*_cat_ for Z-FR-MCA hydrolysis by rCPB2.8 is shown in Fig. 3, in the absence or in the presence of 40 µM heparin. Figs 3A and 3B show the Arrhenius plots of the specificity constant *k*_cat_ */K*_M_ (Fig. 3A) and *k*_cat_ (Fig. 3B) and Figs 3C and 3D show the Eyring plots of the specificity constant *k*_cat_ */K*_M_ (Fig. 3C) and *k*_cat_ (Fig. 3D) in the temperature range from 10 to 55°C. The observed curvature in the plots 3A and 3C is indicative of a change in the rate-limiting step for substrate hydrolysis due to the shift from *k*_2_>> *k*_−1_ at low temperatures and *k*_−1_>> *k*_2_ at high temperatures. The shift is caused by the drastic difference in activation energies for substrate acylation and dissociation when *E*_−1_>> *E*_2_ or *E*_−1_- *E*_2_>>0. On the other hand, the observed linearity in the plots in Figs 3B and 3D is a strong indicative that *k*_3_>>*k*_2_ [42], [56]. Taking together these data, we resolved the kinetic rate constants *k*_1_, *k*_−1_, *k*_2_ and *k*_3_ at the reference temperature (298.15 K), and the thermodynamic parameters *E*_a_, Δ*H,* Δ*S* and Δ*G* for this enzymatic reaction in the absence or in the presence of 40 µM heparin (Table 1).

**Table 1:** Kinetic rate constants of hydrolysis of Z-FR-MCA by rCPB2.8 in absence or in the presence of 40 µM heparin.

ENZYME	Kinetic rate constants (298.15 K)					
rCPB2.8	*k*_1_(mM^−1^.s^−1^)	*k*_−1_ (s^−1^)	*k*_2_ (s^−1^)	*k*_3_ (s^−1^)	*K*_S_ (µM)	*k*_cat_ (s^−1^)
Control	2654±168	1.58±0.03	2.69±0.03	20±2	1.6±0.2	2.4±0.1
40 µM heparin	768±11	0.40±0.01	1.00±0.01	5.7±0.6	1.8±0.2	0.85±0.08

Table 1 shows that the hydrolysis of substrate Z-FR-MCA by rCPB2.8 can be satisfactorily described by a three-step kinetic mechanism, where *K_S_  =  (k*_−*1*_ *+k_2_)/k_1_* stands for the formation of ES complex, *k_2_* stands for the rCPB2.8 acylation step and *k_3_* stands for the rCPB2.8 deacylation step [54]. As expected, the value of *k*_3_ (20±1 s^−1^) was much higher than the value of *k*_2_ (2.7±0.1 s^−1^) and the *k*_cat_ constant is mainly governed by *k*_2_, these values agree with the linearity of the plots (Figs. 3B and 3D), indicating that substrate acylation is rate limiting over the entire temperature range examined. Because *k*_2_ (2.7±0.1 s^−1^) is higher than *k*_−1_ (1.6±0.1 s^−1^), the hydrolysis of Z-FR-MCA substrate by rCPB2.8 can be considered a diffusion controlled process; the constant of specificity *k*_cat_/*K*_M_ (1.7±0.1 µM^−1^.s^−1^) is essentially defined by the association rate constant *k*_1_ (2.7±0.1 µM^−1^.s^−1^). Despite of *k*_3_ >> *k*_2_ and consequently *K*_S_  =  *K*_M_; as *k*_2_ is 1.7-fold higher than *k*_−1,_ *K*_S_ (1.6±0.2 µM) cannot be considered a true dissociation constant.

The association constant *k*_1_ can be limited either by diffusion or conformational rearrangements that facilitate the enzyme-substrate interaction. As diffusion-limited enzyme substrate encounters feature *k*_1_ values > 10^7 ^M^−1^.s^−1^ are linked to small activation energies *E*_1_ < 42 kJ/mol [57], the information on *k*_1_ and *E*_1_ is quite valuable in establishing mechanisms of enzyme-substrate interaction [56]. The present data (Table 2) show that the large value of *E*_1_ (62±5 kJ/mol) and *k*_1_ (2.7 10^6 ^M^−1^.s^−1^) < 10^7 ^M^−1^.s^−1^ in the absence of heparin indicates substantial structural strains linked to the formation of the enzyme-substrate complex. Also, during the formation of enzyme-substrate complex there is a gain of entropy (Δ*S*_1_ =  47±3 J/mol.K) and it indicates that rCPB2.8 can assume a more open conformation [45]. As the entropy of activation is negative for the acylation and deacylation step [45], a substantial loss of entropy is observed during the acylation step as expected (Δ*S*_2_ =  –75±3 J/mol.K) - strongly suggesting a refolding of the enzyme [45]. The “stickiness” of substrate, defined as the rate at which a substrate reacts to give products relative to the rate at which that substrate dissociates, in the absence of heparin *k*_2_/*k*_−1_  =  2.0±0.2, shows that the propensity of the ES complex to undergo acylation is 2.0-fold larger than its propensity to decouple in E + S forms. The large positive nature of the term Δ*G*_−1_ – Δ*G*_2_  =  161±7 kJ/mol signals a substantial difference in the energetic cost of dissociating the substrate compared with acylating it.

**Table 2:** Activation energies, entropies, enthalpies and Gibbs free energies of hydrolysis of Z-FR-MCA by rCPB2.8 in absence or in the presence of 40 µM heparin.

	Activation Energies (kJ.mol^−1^)			Entropies (J.mol.K^−1^)			Enthalpies (kJ.mol^−1^)			Gibbs Free Energies (kJ.mol^−1^)		
rCPB2.8	E_1_	E_−1_	E_2_	ΔS_1_	ΔS_−1_	ΔS_2_	ΔH_1_	ΔH_−1_	ΔH_2_	ΔG_1_	ΔG_−1_	ΔG_2_
Control	62±5	144±12	53±4	47±3	–309±19	–75±3	59±1	142±5	51±2	45±4	234±15	73±6
40 µM heparin	34±3	113±10	51±4	–84±6	–452±25	–83±4	32±2	112±6	49±2	57±5	247±17	74±7

Interestingly, the presence of heparin induces a large effect in kinetic constants of the enzyme (Table 1). The data analysis revealed that the presence of 40 µM heparin affects all steps of the reaction: (i) it decreases 3.5-fold the *k*_1_ and reduces 4.0-fold the *k*_−1_, (ii) it affects the acyl-enzyme accumulation with pronounced decrease in *k*_2_ (2.7-fold), and also decrease in *k*_3_ (3.5-fold). The effect of heparin on *k*_1_ is characterized by a large enthalpy change of Δ*H*_control_ - Δ*H*_heparin_  =  27 kJ/mol that is compensated by a large entropy gain Δ*S*_control_ - Δ*S*_heparin_  =  143 J/mol/K (Table 2).

### The influence of Heparin upon rCPB2.8 pH Activity Profiles

**Figure 4:**
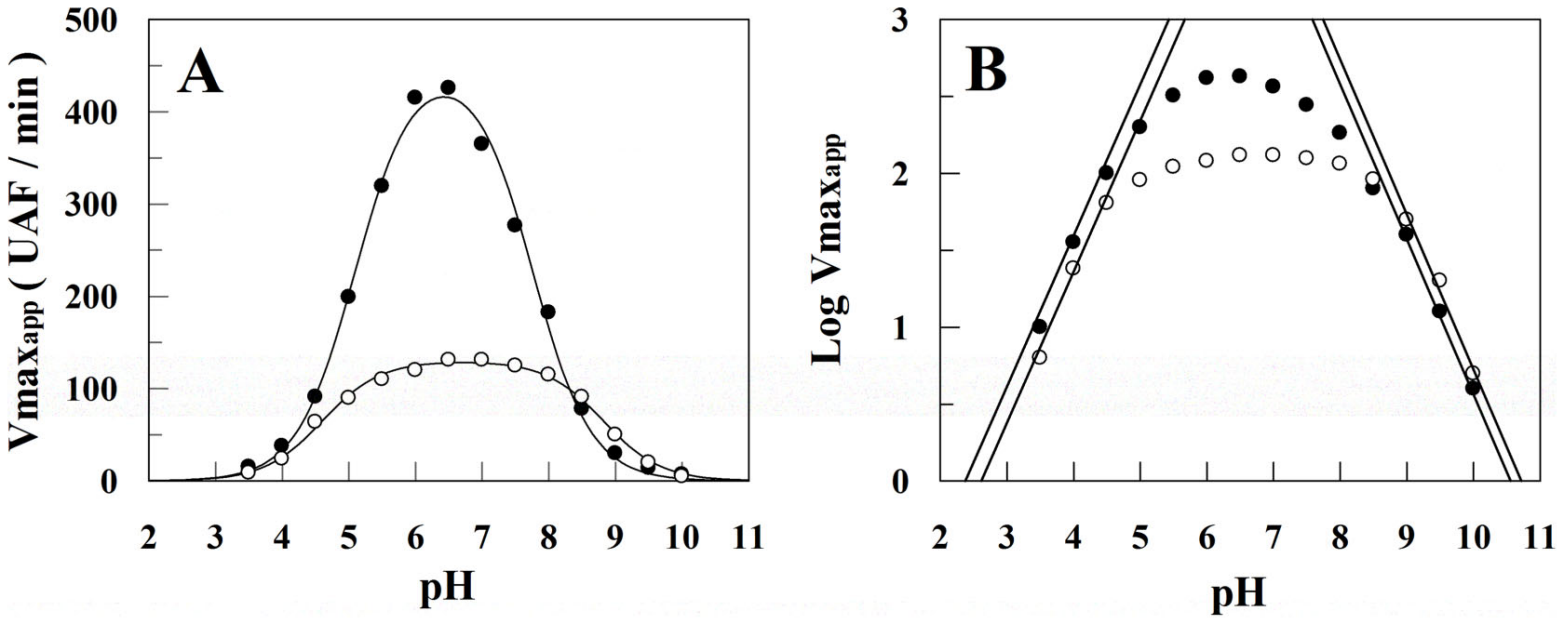
The influence of heparin upon rCPB2.8 pH activity profiles . The substrate Z-FR-MCA hydrolysis was performed in absence (•–•) and in presence (○–○) of 40 µM heparin at 35°C in universal buffer containing 25 mM glycine, 25 mM acetic acid, 25 mM Mes and 75 mM Tris, containing 20% glycerol and 5 mM DTT, the pH of buffers were adjusted using HCl or NaOH diluted solutions as described under “Methods.” *A*, the pH activity profiles data were fitted according to Equation 10 by using non-linear regression software system. The associated table shows the values of p*K*_ES1_ and p*K*_ES2_ in absence and in the presence of heparin. *B*, Dixon-Web plot of the log *V*_maxapp_ versus pH where the dot lines of slope  =  1 and slope  =  -1 tangent to the curve.

The effect of heparin on the pH activity profiles of rCPB2.8 was analyzed by monitoring the enzyme-catalyzed hydrolysis of the Z-FR-MCA substrate. Fig 4A shows that enzyme displays bell-shaped pH dependence both in the absence and presence of heparin. Fig 4B shows the Dixon-Web plot of log *V*_maxapp_ versus pH where the dot lines of slope  =  1 and slope  =  -1 tangent to the curve at very low log *V*_maxapp_ values intersect the horizontal log *V*_max_ line at p*K*_ES1_ and p*K*_ES2_, respectively. When rCPB2.8 was assayed in the presence of heparin a large effect of heparin was observed upon the pH-activity profile. Basically, 40 μM heparin promoted a general decreases of about 3.5-fold in the values of *V*_max_ observed for Z-FR-MCA hydrolysis and shifted the rCPB2.8 pH activity profile about 0.3 units to the right. Table 3 shows that in the presence of heparin the value of p*K*_ES1_ was shifted from 5.1 to 4.6, the p*K*_ES2_ was largely increased from 7.8 to 8.8 and the pH_opt_ of rCPB2.8 was increased from 6.4 to 6.7.

**Table 3:** pK_ES_ and pH_opt_ values obtained by the monitoring the hydrolysis of Z-FR-MCA by the enzyme rCPB2.8.in absence or in the presence of 40 µM heparin.

	Control	Heparin 40 µM
p*K*_ES1_	5.1±0.1	4.6± 0.1
p*K*_ES2_	7.8±0.1	8.8±0.1
p*K*_ES1_	6.4±0.1	6.7±0.1

The acidic p*K*_ES1_ values stands to deprotonation of the active Cys^25^, while the basic p*K*_ES2_ values results from deprotonation of His^163^ in the presence of Z-FR-MCA substrate at the active site of the enzyme. Taken together, these data strongly suggest that heparin is altering the ionization of catalytic (Cys^25^)-S^−^/(His^163^)-Im^+^ H ion pair of the rCPB2.8. A change in the ionization of the catalytic (Cys^25^)-S^−^/(His^163^)-Im^+^ H ion pair may be related to conformational change, or to direct electrostatic modulation induced by heparin binding or both [58], [59].

### Heparin Binding-Induced rCPB2.8 Conformational Change

**Figure 5:**
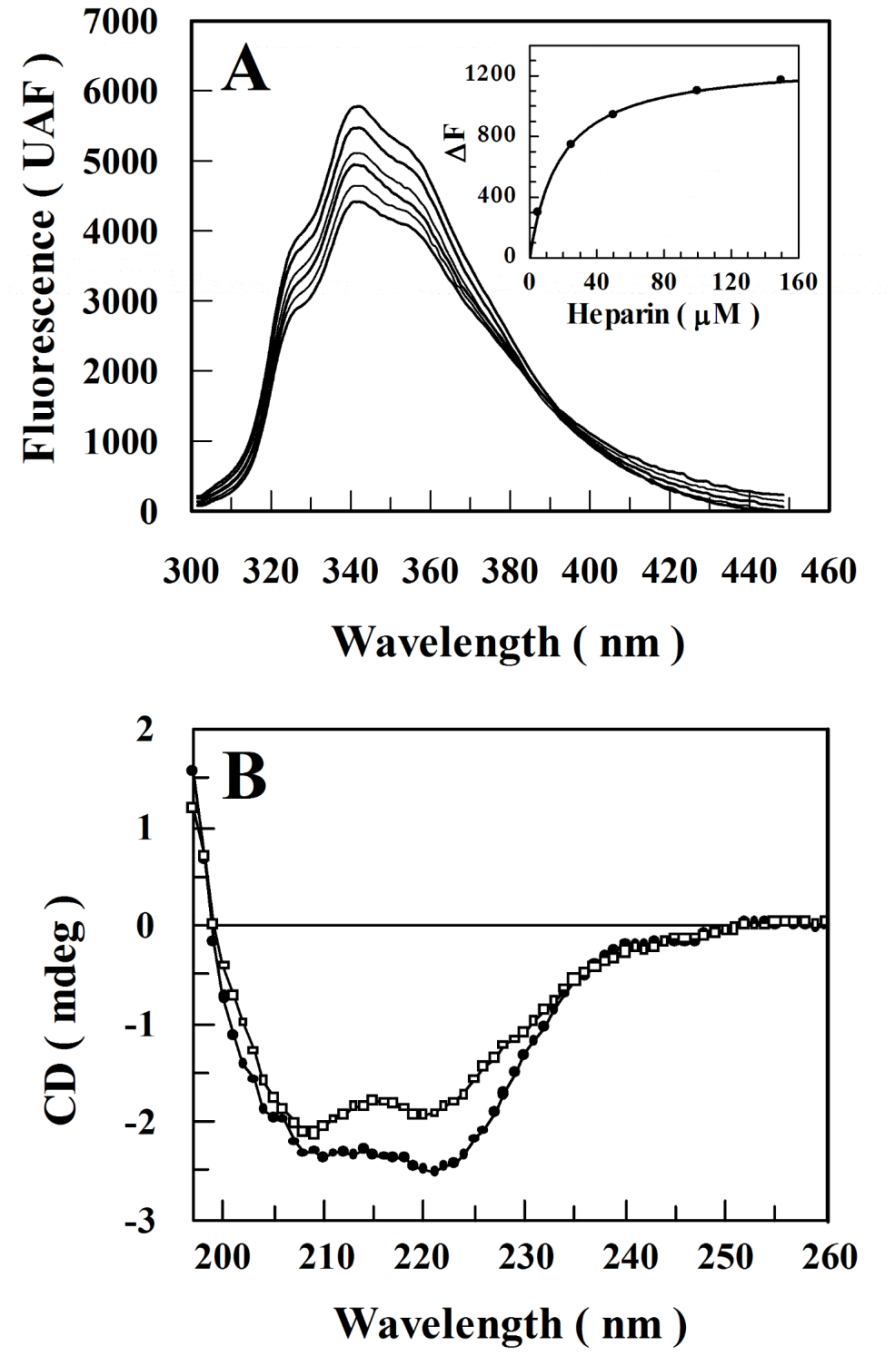
Heparin binding-induced rCPB2.8 conformational change. *A*, the intrinsic fluorescence of tryptophan residues of the rCPB2.8 was monitored in 50 mM sodium phosphate buffer containing 20% glycerol at 35 °C by measuring the emission of fluorescence between at 300 — 450 nm after excitation at λ_ex_  =  290 nm in the absence or in the presence of different concentrations of heparin (0 — 167 µM). The insert in *A* shows the variation of the intrinsic fluoresce emission ΔF (F-F_0_) as a function of heparin concentration. *B*, effects of heparin on rCPB2.8 circular dichroism spectra. About 2 µM rCPB2.8 were determined in 5 mM sodium phosphate buffer pH 5.8 in the absence (•–•) or in the presence (□–□) of 40 µM heparin.

The conformational change in rCPB2.8 induced by heparin binding was assessed by monitoring changes in intrinsic tryptophan fluorescence [60]. Fig 5A shows the changes in the rCPB2.8 fluorescence emission spectra as a function of heparin concentration, increasing the concentration of heparin resulted in a progressive decrease in fluorescence emission spectra of the enzyme with an isoemissive point around 390 nm. The presence of an isoemissive point must be observed if there are two emissive species, irrespective of the origin of the species or their kinetics [61]. In this case, the isoemissive point is related to the presence of both forms of enzyme: free enzyme (E) and enzyme-heparin complex (EH). Therefore, the variation of 1.0 μM rCPB2.8 fluorescence in function of heparin concentration was fitted according to Eq. 11. The kinetic analysis shows that heparin bind rCPB2.8 by a saturable bimolecular reaction with an apparent *K*_d_ of 16±2 µM (Fig. 5A, *insert*).

The effect of heparin on rCPB2.8 conformation was also analyzed by CD spectroscopy. Fig 5B shows that the presence of 40 μM heparin causes a significant change in the spectral envelope of the rCPB2.8, leading a positive increase of the ellipticity value at [θ]_222_ nm, suggesting that heparin decreases the helicity of rCPB2.8. Indeed, Table 4 exhibits the secondary structure content of enzyme in the absence or in presence of 40 µM heparin concentration. The fractions of the different rCPB 2.8 structural types, α, β, and remainder (R), were computed from the CD spectra (197–260 nm) as described previously (38). The data show a large decrease of 27% in the rCPB2.8 α-helix content induced by heparin, whereas β-structure and remainder content increased. These changes are likely to reflect the enzyme-heparin interaction.

**Table 4:** Far UV (197 – 260 nm) CD spectra of rCPB2.8.

	α-Helix %	β-Sheet %	Remaining %
rCPB2.8 – pH 5.0	18±1	38±1	44±1
rCPB2.8 + 100 µM Heparin	13±1	43±1	44±1

The CD analysis of rCPB2.8 was proceeded at pH 5.0 in the absence or in the presence of 40 µM heparin as describe under “Methods.”

### Heparin Decreases the Rate of Inhibition of rCPB2.8 by its N-terminal Pro-Region

CPB2.8 and other lysosomal cysteine proteases are synthesized as inactive zymogens due to the presence of an *N*-terminal pro-region; this domain is a potent inhibitor of the cysteine proteases [62], [63]. Cleavage and dissociation of the *N*-terminal propeptide domain with concomitant activation of the cysteine protease occur as the result of an autocatalytic processing under acidic conditions [64] or in the presence of glycosaminoglycans [65]–[67].

**Figure 6:**
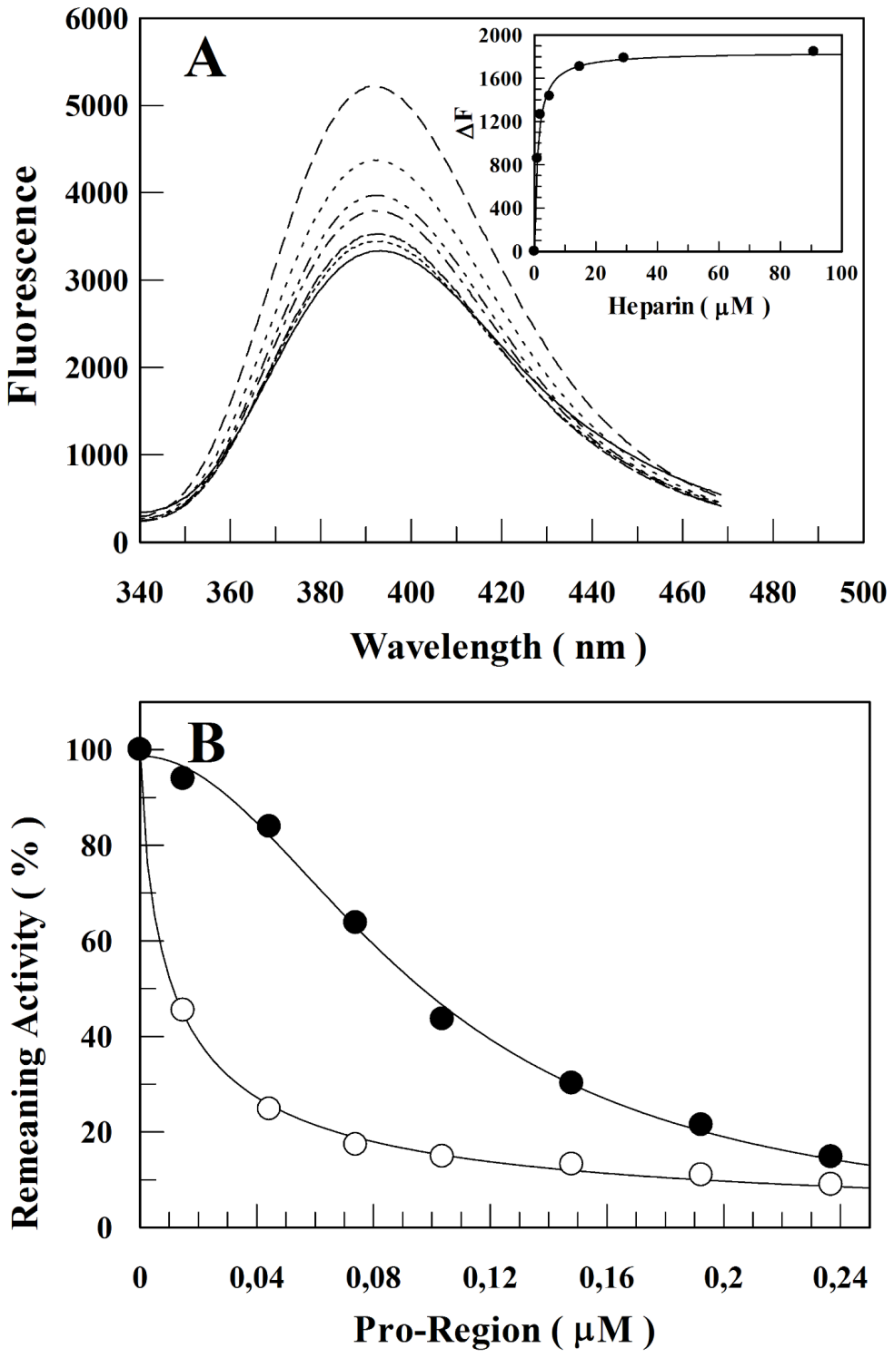
Effects of heparin on the *N*-terminal pro-region of rCPB2.8. *A*, the influence of heparin concentration upon *N*-terminal pro-region segment intrinsic fluorescence emission. The insert in *A* shows the variation of the intrinsic fluoresce emission ΔF (F-F_0_) of the *N*-terminal pro-region as a function of heparin concentration (0 — 167 µM) as described under “Methods.” *B*, heparin prevents the inhibitory activity of *N*-terminal pro-region upon rCPB2.8. The remaining activity of the enzyme was plotted as a function of *N*-terminal pro-region concentration in the absence (○–○) or in the presence (•–•) of 40 µM heparin.

The ability of heparin to bind rCPB2.8 *N*-terminal pro-region was analyzed by measuring the changes in the intrinsic fluorescence of the pro-region as a function of heparin concentration (Fig. 6A). Heparin also promoted a concentration-dependent decrease on the intrinsic fluorescence emission spectra of the pro-region. Heparin interacts with *N*-terminal pro-region of rCPB2.8 with a dissociation constant of 1.5±0.1 µM.

Heparin binding Cardin-motifs (B-X-B-B-X or B-B-X-X-B-B-B, where B is a basic amino acid residue and X a hidropathic residue) on the *N*-terminal pro-region of human procathepsin L and of *Schistosoma mansoni* (*Sm*) procathepsin B mediate zymogen destabilization and its subsequence activation [66], [67]. Also, GAGs facilitate human procathepsin B activation through disruption of proregion-mature enzyme interactions binding in the several positively charged residues in the propeptide [68]. Therefore, the low affinity of heparin binding to mature human cathepsin L, mature *Sm* cathepsin B, and mature human cathepsin B are related to Cardin binding site and charged residues that is located at the propeptide region, respectively, which is removed during zymogen auto-processing. Our results shown that heparin binding proregion of rCPB2.8 with *K*_d_ =  1.5±0.1 µM (Fig 6A). On the other hand, heparin binding to mature form of the enzyme with *K*_d_ =  16±2 µM (Fig. 5A). Taking together, heparin has about 10-fold more affinity to proenzyme rCPB2.8 than to mature form.

Fig. 6B reports the inhibition of rCPB2.8 by its *N*-terminal pro-region in the absence or in the presence of heparin. The data show that, in the absence of heparin, the pro-region drove a powerful inhibition of rCPB2.8 as expected. The presence of heparin dislodged the inhibitory curve of pro-region to the right. The dissociation constant of inhibition was found to be 1.0±0.1 nM, a value that is about 10-fold lower than its dissociation constant measured in the presence of heparin (*K*_app_  =  10±1 nM). It is likely that the binding of heparin to both rCPB2.8 and pro-region accounts for the large decrease in the inhibition rate.

## Discussion

The presence of heparin in the rCPB2.8 kinetic assays results in a decreased *V*_max_ (*k*_cat_) value for the hydrolysis of Z-FR-MCA without change the substrate dissociation constant (Fig. 2). Heparin inhibits rCPB2.8 endopeptidase activity upon the substrate Z-FR-MCA by an apparent linear intersecting non-competitive type inhibition fashion, where heparin and the fluorogenic substrate Z-FR-MCA bind reversible, randomly, and independently at different sites of the enzyme resulting in inactive ternary ESI complex (Figure 1). It was observed that heparin binds free rCPB2.8 (E) with a dissociation constant of *K*_H_  =  17±1 µM; and heparin binds the complex enzyme/substrate (ES) with the same dissociation constant value (α = 1.0). These data strongly suggest that heparin binding distorts the structure of rCPB2.8 sufficiently to prevent the catalysis of the ternary complex ESI.

Although the three-step mechanism for cysteine cathepsins-catalyzed hydrolysis of peptides is widely accepted [54]–[55], the individual rate constants that describe the steps have not been determined in the studies of rCPB2.8’s inhibition. More, if *k*_2_ contributes significantly to the *K*_S_  =  (*k*_−1_ + *k*_2_)/*k*_1_ relationship, then an inhibitor that affects the *k*_cat_  =  *k*_2_.*k*_3_/*k_2_*+*k_3_* value may also affect the *K*_S_ value. Consequently, the only way to get an accurate picture of rCPB2.8 inhibition by heparin is through the determination of the effects of heparin upon each individual rate constant *k*_1_, *k*_−1_, *k*_2_ and *k*_3_.

In order to determine the values for the kinetic constants *k*_1_, *k*_−1_, *k*_2_ *and k*_3_ of the Z-FR-MCA hydrolysis by rCPB2.8, we studied the effect of temperature dependence upon *k*_cat_ and *k*_cat_/*K*_M_ for the enzyme inhibition (Fig. 3). As expected, heparin affected the acyl-enzyme formation with pronounced decrease in *k*_2_ (2.7-fold), and also decreased the rate of the hydrolysis of the acyl-enzyme complex *k*_3_ 3.5-fold (Table 1). Interesting, the magnitude of the effect of heparin on the substrate association rate constant step (*k*_1_) was basically the same as the heparin-induced decrease the substrate dissociation constant step (*k*_−1_+ *k*_2_); in both cases heparin decreased 3.5-fold its values (Table 1). In other words, the apparent dissociation constant of the substrate *K*_S_ was the same in the absence (1.6±0.2 µM) or in the presence of heparin (1.8±0.2 µM). These data corroborate the previously results depicted in Fig. 2 which describe heparin as an apparent non-competitive inhibitor of the rCPB2.8.

The binding of heparin with rCPB2.8 was characterized by a large enthalpy-entropy energetic compensation (Table 2), the data proves that heparin decreases diffusion of Z-FR-MCA into the rCPB2.8 active site by 3.5-fold and that this effect is linked to significant conformational change that increases the energetic barrier for formation of the productive ES complex by Δ*G  = * 12 kJ/mol. As expected, heparin also increases the energetic barrier for the substrate dissociation from the ES complex by decreasing 4.0-fold the dissociation rate *k*_−1_. In the presence of heparin the energetic cost (Δ*G*_−1_ – Δ*G*_2_) is also increased 13 kJ/mol. Both large values of Δ*G* of the association and dissociation steps indicate substantial structural strains linked to the formation/dissociation of the ES complex in the presence of heparin, which underscore a conformational change that prevents the diffusion of substrate in the rCPB2.8 active site.

The binding of heparin to rCPB2.8 also perturbs its intrinsic fluorescence. The binding of heparin induced a saturable suppression upon the intrinsic fluorescence emission spectra of the enzyme (Fig. 5A). Interestingly, the estimated affinity of heparin for rCPB2.8 measured by intrinsic fluorescence assays (*K*_d_  =  16±2 µM) was very similar to the values of *K*_I_ found for the inhibition of its endopeptidase activity (*K*_I_  =  17±1 µM). These results indicate that heparin binding is perturbing the rCPB2.8 structure in a similar manner.

The quenching in the intrinsic fluorescence emission spectra of the enzyme indicates that the buried tryptophan residues became exposed to a more polar environment in the presence of heparin [60]. Because rCPB2.8 contains numerous tryptophan residues, the change in fluorescence could not be ascribed to specific residues. It is important to note that rCPB2.8 has 8 tryptophan residues in its composition [22], and that heparin binding produced a decrease of 25% in the intrinsic fluorescence of tryptophan residues present in it. Interesting, the docking analysis show that the interaction of heparin with Trp^185^ at subsite S_1_′ and with Trp^189^ residues at subsite S_2_’ of the rCPB2.8 active site suppresses the intrinsic fluorescence of rCPB2.8. The present data are consistent with a conformational change induced by heparin binding that affects both enzyme activity and the intrinsic fluorescence of tryptophan residues.

Binding to heparin also significantly decreases the α-helix content of rCPB2.8. This binding was marked by significant changes in the shape and position of the CD spectral envelope (Fig. 5B). Table 4 shows that heparin decreases the helical content of rCPB2.8 by decreasing the number of amino acids residues in the helical conformation; and this effect seems to be related to the increase of the β-sheet content. These data strongly suggest that the conformational change induced by heparin binding leads to a decrease in the catalysis of the rCPB2.8 for the substrate Z-FR-MCA.

The presence of heparin disturbed the pH activity profile of the enzyme (Fig. 4), in the presence of heparin the value of the p*K*_ES1_ was shifted from 5.1 to 4.6, and the p*K*_ES2_ was increased from 7.8 to 8.8 (Table 3). For members of the papain family, the ionic thiolate-imidazole (Cys^25^)-S− by (His^159^)-Im+H interaction of the active site is predicted to result in low nucleophilic reactivity of the ion pair S atom as in the case for an un-ionized thiol group. It has been suggested that the release of nucleophilic reactivity in Cys^25^ requires decrease in the solvation of (Cys^25^)-S− by (His^159^)-Im+H occasioned by movement of the cleft around Trp^177^ (papain numbering). This Trp^177^ movement results in His^159^ being exposed to solvent with consequent decrease in its solvation of the thiolate anion of Cys^25^. More, the Trp^177^ movement is correlated with the interruption of Trp^177^-Gln^19^ hydrogen bond across p*K*_a_ 4; the rupture of the hydrogen bond between Trp^177^-Gln^19^ seems to be essential to establish the oxyanion hole in binding the developing tetrahedral species during the acylation process [58], [59]. In rCPB2.8, the function of Trp^177^ residue of papain in catalysis is assumed by Trp^185^ [29].

Recently, it has been demonstrated that heparin and several other GAGs can increase the rate of pro-cysteine cathepsins maturation by disrupting the interactions between its *N-*terminal pro-region and mature enzymes [22], [65], [68]. Indeed, our results clearly show that heparin interacts with the *N*-terminal pro-region of rCPB2.8 showing dissociation constant of 1.5±0.1 µM, a value that is about 10-fold lower than its dissociation constant for mature rCPB2.8 (*K*_d_  =  16±2 µM). The results strongly suggest that heparin interacts preferentially with the inactive pro-enzyme rather than its mature form. It is also important to mention that the interaction of heparin with the *N*-terminal pro-region of rCPB2.8 significantly decreased its inhibitory activity against the mature enzyme (Fig. 6B). Despite of the exact molecular mechanism for rCPB2.8 zymogen activation is unknown yet, the procathepsin B activation shows that this mechanism involves unimolecular conformational change followed by a bimolecular proteolytic removal of the propeptide, which can be accomplished in one or more steps. This activation is also facilitated by glycosaminoglycans or by binding to negatively charged surfaces [65].

Taken together, the present results suggest that heparin, at low concentrations (below 2 µM), can both weaken the effectiveness of the *N*-terminal pro-region inhibitor and potentiate the conversion of pro-enzyme into mature CPB form and, conversely, higher concentrations of heparin (above 20 µM) can inhibit this conversion. The large amounts of proteoglycans and free chains of GAG present in the extracellular matrix can overcome the high rate of conversion pro-enzyme into mature forms of CPB2.8, thereby decreasing the amount of the active enzyme present.

Thus the results suggest that GAGs from the ECM of host cells may be important binding sites for the parasite cysteine proteases. In the ECM, GAGs are covalently bound to core proteins, forming a dense network of fixed negative charges available for interaction with CPB enzymes released extracellularly by *Leishmania* during its process of infection. It has been shown that *Leishmania* can secrete cysteine protease enzymes (CPB) to the ECM of host cells; this process is related to parasite infection by proteolysis of fibronectin from host ECM [37]. Also, it is well known that during ECM remodeling or pathological degradation mediated by several proteolytic enzymes, GAG chains are released following hydrolysis of core proteins [69], [70].

GAG chains released from ECM proteoglycans by the action of proteolytic enzymes during parasite infection and inflammatory response may contribute to the regulation of CPB enzymes by themselves and in association with inhibitors such as its *N*-terminal pro-region. So, depending on their concentration, GAG can either stimulate or antagonize the activity of CPB enzymes during parasite infection. Similar GAG control has also been described for other classes of proteolytic enzymes [71].

## References

[1] Sajid M, McKerrow JH (2002) Cysteine proteases of parasitic organisms. Mol Biochem Parasitol 120: 1-21. Review. Erratum in: Mol Biochem Parasitol 121: 159. 10.1016/s0166-6851(01)00438-8 11849701

[2] Coombs GH (1982) Proteinases of *Leishmania mexicana* and other flagellate protozoa. Parasitology 84: 149–155. 6460959 10.1017/s003118200005174x

[3] Pupkis MF, Coombs GH (1984) Purification and characterization of proteolytic enzymes of *Leishmania mexicana* amastigotes and promastigotes. J Gen Microbiol 130: 2375–2383. 6389769 10.1099/00221287-130-9-2375

[4] Robertson CD, Coombs GH (1990) Characterization of three groups of cysteine proteinases in the amastigotes of *Leishmania mexicana mexicana* . Mol Biochem Parasitol 42: 269–276. 2270108 10.1016/0166-6851(90)90170-q

[5] Robertson CD, Coombs GH (1992) Stage-specific proteinases of *Leishmania mexicana* promastigotes. FEMS Microbiol Lett 94: 127–132. 10.1111/j.1574-6968.1992.tb05301.x 1521760

[6] Bates PA, Robertson CD, Coombs GH (1994) Expression of cysteine proteinases by metacyclic promastigotes of *Leishmania mexicana* . J Euk Microbiol 41: 199–203. 8049682 10.1111/j.1550-7408.1994.tb01497.x

[7] Mottram JC, Frame MC, Brooks DR, Tetley L, Hutchison JE, et al (1997) The multiple *cpb* cysteine proteinase genes of *Leishmania mexicana* encode isoenzymes that differ in their stage regulation and substrate preferences. J Biol Chem 272: 14285–14293. 9162063 10.1074/jbc.272.22.14285

[8] Souza AE, Waugh S, Coombs GH, Mottram JC (1992) Characterization of a multicopy gene for a major stage-specific cysteine proteinase of *Leishmania mexicana* . FEBS Lett 311: 124–127. 1397299 10.1016/0014-5793(92)81382-v

[9] Robertson CD, Coombs GH (1994) Multiple high activity cysteine proteases of *Leishmania mexicana* are encoded by the *lmcpb* gene array. Microbiology 140: 417–424. 8180705 10.1099/13500872-140-2-417

[10] Mottram JC, Souza AE, Hutchison JE, Carter R, Frame MJ, et al (1996) Evidence from disruption of the *lmcpb* gene array of *Leishmania mexicana* that cysteine proteinases are virulence factors. Proc Natl Acad Sci USA 93: 6008–6013. 8650210 10.1073/pnas.93.12.6008 PMC39179

[11] Coombs GH, Mottram JC (1997) Proteinases of trypanosomes and *Leishmania*. In: Hide, G, Mottram, JC, Coombs, GH, Holmes, PH, editors. Trypanosomiasis and leishmaniasis: biology and control. Oxford: Oxford-CAB International. pp. 177–197.

[12] Traub-Cseko YM, Duboise M, Boukai LK, McMahon-Pratt D (1993) Identification of two distinct cysteine proteinase genes of *Leishmania pifanoi* axenic amastigotes using the polymerase chain reaction. Mol Biochem Parasitol 57: 101–116. 8426606 10.1016/0166-6851(93)90248-v

[13] Sakanari JA, Nadler SA, Chan VJ, Engel JC, Leptak C, et al (1997) *Leishmania major*: comparison of the cathepsin L- and B-like cysteine protease genes with those of other trypanosomatids. Exp Parasitol 85: 63–76. 9024203 10.1006/expr.1996.4116

[14] Campetella O, Henriksson J, Åslund L, Frasch ACC, Pettersson U, et al (1992) The major cysteine proteinase (cruzipain) from *Trypanosoma cruzi* is encoded by multiple polymorphic tandemly organized genes located on different chromosomes. Mol Biochem Parasitol 50: 225–234. 1311053 10.1016/0166-6851(92)90219-a

[15] Murta ACM, Persechini PM, Padron TS, Souza W, Guimarães JA, et al (1990) Structural and functional identification of GP57/51 antigen of *Trypanosoma cruzi* as a cysteine proteinase. Mol Biochem Parasitol 43: 27–38. 1705310 10.1016/0166-6851(90)90127-8

[16] Mottram JC, North MJ, Barry JD, Coombs GH (1989) A cysteine proteinase cDNA from *Trypanosoma brucei* predicts an enzyme with an unusual *C*-terminal extension. FEBS Lett 258: 211–215. 2599086 10.1016/0014-5793(89)81655-2

[17] Coombs GH, Mottram JC (1997) Parasite Proteinases and Amino Acid Metabolism: Possibilities for Chemotherapeutic Exploitation. Parasitology 114: S61–S80. 9309769

[18] DeSouza LS, Lang T, Prina E, Hellio R, Antoine JC (1995) Intracellular *Leishmania amazonensis* Amastigotes Internalize and Degrade MHC Class II Molecules of Their Host Cells. J Cell Sci 108: 3219–3231. 7593283 10.1242/jcs.108.10.3219

[19] Alexander J, Coombs GH, Mottram JC (1998) *Leishmania mexicana* Cysteine Proteinase- Deficient Mutants Have Attenuated Virulence for Mice and Potentiate a Th1 Response. J Immunol161: 6794–6801. 9862710

[20] Barrett MP, Mottram JC, Coombs GH (1999) Recent advances in identifying and validating drug targets in trypanosomes and leishmanias. Trends Microbiol 7: 82–88. 10081086 10.1016/s0966-842x(98)01433-4

[21] Caffrey CR, Scory S, Steverding D (2000) Cysteine proteinases of trypanosome parasites: novel targets for chemotherapy. Curr Drug Targets 1: 155–162. 11465068 10.2174/1389450003349290

[22] Sanderson SJ, Pollock KG, Hilley JD, Meldal M, St Hilaire PM, et al (2000) Expression and characterization of a recombinant cysteine proteinase of *Leishmania mexicana* . Biochem J 347: 383–388. 10749667 10.1042/0264-6021:3470383 PMC1220970

[23] Alves LC, Melo RL, Sanderson SJ, Mottram JC, Coombs GH, et al (2001) S1 subsite specificity of a recombinant cysteine proteinase, CPB, of *Leishmania Mexicana* compared with cruzain, human cathepsin L and papain using substrates containing non-natural basic amino acids. Eur J Biochem 268: 1206–1212. 11231271 10.1046/j.1432-1327.2001.01973.x

[24] Alves LC, Judice WA, St Hilaire PM, Meldal M, Sanderson SJ, et al (2001) Substrate specificity of recombinant cysteine proteinase, CPB, of *Leishmania mexicana* . Mol Biochem Parasitol 116: 1–9. 11463460 10.1016/s0166-6851(01)00290-0

[25] Alves LC, Melo RL, Cezari MH, Sanderson SJ, Mottram JC, et al (2001) Analysis of the S2 subsite specificities of the recombinant cysteine proteinases CPB of *Leishmania mexicana*, and cruzain of *Trypanosoma cruzi*, using fluorescent substrates containing non-natural basic amino acids. Mol Biochem Parasitol 117: 137–143. 11606223 10.1016/s0166-6851(01)00340-1

[26] St Hilaire PM, Alves LC, Sanderson SJ, Mottram JC, Juliano MA, et al (2000) The substrate specificity of a recombinant cysteine protease from *Leishmania mexicana*: application of a combinatorial peptide library approach. Chem Biol Chem 1: 115–122. 10.1002/1439-7633(20000818)1:2<115::aid-cbic115>3.3.co;2-# 11828405

[27] Juliano MA, Brooks DR, Selzer PM, Pandolfo HL, Judice WA, et al (2004) Differences in substrate specificities between cysteine protease CPB isoforms of *Leishmania mexicana* are mediated by a few amino acid changes. Eur J Biochem 271: 3704–3714. 15355348 10.1111/j.1432-1033.2004.04311.x

[28] Alves LC, St Hilaire PM, Meldal M, Sanderson SJ, Mottram JC, et al (2001) Identification of peptides inhibitory to recombinant cysteine proteinase, CPB, of *Leishmania mexicana* . Mol Biochem Parasitol 114: 81–88. 11356516 10.1016/s0166-6851(01)00239-0

[29] St Hilaire PM, Alves LC, Herrera F, Renil M, Sanderson S, et al (2002) Solid-Phase library synthesis, screening and selection of tight-binding reduced peptide bond inhibitors of a recombinant *Leishmania mexicana* cysteine protease B. J Med Chem. 45: 1971–1982. 10.1021/jm0110901 11985465

[30] Graven A, St Hilaire PM, Sanderson SJ, Mottram JC, Coombs GH, et al (2001) Combinatorial library of peptide isosters based on Diels-Alder reactions: identification of novel inhibitors against a recombinant cysteine protease from *Leishmania mexicana* . J Comb Chem 3: 441–452. 11549362 10.1021/cc0001102

[31] Stoka V, McKerrow JH, Cazzulo JJ, Turk V (1998) Substrate inhibition of cruzipain is not affected by the C-terminal domain. FEBS Lett 429: 129–133. 9650575 10.1016/s0014-5793(98)00532-8

[32] Conrad HE (1998) Heparin-Binding Proteins. Academic Press Inc., New York.

[33] Del Nery E, Juliano MA, Lima APCA, Scharfstein J, Juliano L (1997) Kininogenase activity by the major cysteinyl proteinase (cruzipain) from Trypanosoma cruzi. J Biol Chem 272: 25713–25718. 9325296 10.1074/jbc.272.41.25713

[34] Bartlett AH, Park PW (2010) Proteoglycans in host-pathogen interactions: molecular mechanisms and therapeutic implications. Expert Rev Mol Med 12: e5. 20113533 10.1017/S1462399409001367 PMC4634875

[35] Love DC, Esko JD, Mosser DM (1993) A heparin-binding activity on Leishmania amastigotes which mediates adhesion to cellular proteoglycans. J Biol Chem 123: 759–766. 10.1083/jcb.123.3.759 PMC2200127 8227137

[36] de Castro Côrtes LM, de Souza Pereira MC, da Silva FS, Pereira BA, de Oliveira Junior FO, et al (2012) Participation of heparin binding proteins from the surface of *Leishmania (Viannia) braziliensis* promastigotes in the adhesion of parasites to *Lutzomyia longipalpis* cells (Lulo) in vitro. Parasit Vectors 5: 142. 22805335 10.1186/1756-3305-5-142 PMC3419669

[37] Kulkarni MM, Jones EA, McMaster WR, McGwire BS (2008) Fibronectin binding and proteolytic degradation by Leishmania and effects on macrophage activation. Infect Immun 76: 1738–1747. 18212076 10.1128/IAI.01274-07 PMC2292850

[38] Almeida PC, Nantes IL, Rizzi CCA, Judice WAS, Chagas JR, et al (1999) Cysteine proteinase activity regulation. A possible role of heparin and heparin-like glycosaminoglycans. J Biol Chem 274: 30433–30438. 10521421 10.1074/jbc.274.43.30433

[39] Almeida PC, Nantes IL, Chagas JR, Rizzi CCA, Faljoni-Alário A, et al (2001) Cathepsin B activity regulation. Heparin-like glycosaminogylcans protect human cathepsin B from alkaline pH-induced inactivation. J Biol Chem 276: 944–951. 11016923 10.1074/jbc.M003820200

[40] Lima APCA, Almeida PC, Tersariol ILS, Schmitz V, Schmaier AH, et al (2002) Heparan sulfate modulates kinin release by Trypanosoma cruzi through the activity of cruzipain. J Biol Chem 277: 5875–5881. 11726662 10.1074/jbc.M108518200

[41] Li Z, Hou WS, Bromme D (2000) Collagenolytic activity of cathepsin K is specifically modulated by cartilage-resident chondroitin sulfates. Biochemistry 39: 529–536. 10642177 10.1021/bi992251u

[42] Ayala YM, Di Cera E (2000) A simple method for the determination of individual rate constants for substrate hydrolysis by serine proteases. Protein Sci 9: 1589–1593. 10975580 10.1110/ps.9.8.1589 PMC2144722

[43] Judice WA, Cezari MH, Lima AP, Scharfstein J, Chagas JR, et al (2001) Comparison of the specificity, stability and individual rate constants with respective activation parameters for the peptidase activity of cruzipain and its recombinant form, cruzain, from Trypanosoma cruzi. Eur J Biochem 268: 6578–6586. 11737212 10.1046/j.0014-2956.2001.02612.x

[44] Judice WA, Mottram JC, Coombs GH, Juliano MA, Juliano L (2005) Specific negative charges in cysteine protease isoforms of Leishmania mexicana are highly influential on the substrate binding and hydrolysis. Mol Biochem Parasitol 144: 36–43. 16125801 10.1016/j.molbiopara.2005.07.004

[45] Laidler KJ, Peterman BF (1979) Temperature effects in enzyme kinetics. Methods in Enzymology 63: 234–257. 502860 10.1016/0076-6879(79)63012-4

[46] Cornish-Bowden A (1999) Fundaments of Enzyme Kinetics. Portland Press, London, UK.

[47] Polgar L (1999) Oligopeptidase B: a new type of serine peptidase with unique substrate-dpendent temperature sensitivity. Biochemistry 38: 15548–15555. 10569938 10.1021/bi991767a

[48] Pimenta DC, Nantes IL, de Souza ES, Le Bonniec B, Ito AS, et al (2002) Interaction of heparin with internally quenched fluorogenic peptides derived from heparin-binding consensus sequences, kallistatin and anti-thrombin III. Biochem J 366: 435–446. 12000310 10.1042/BJ20020023 PMC1222784

[49] Shinjo SK, Tersariol IL, Oliveira V, Nakaie CR, Oshiro ME, et al (2002) Heparin and heparan sulfate disaccharides bind to the exchanger inhibitor peptide region of Na+/Ca2+ exchanger and reduce the cytosolic calcium of smooth muscle cell lines. Requirement of C4-C5 unsaturation and 1 4 glycosidic linkage for activity. J Biol Chem 277: 48227–48233. 12374809 10.1074/jbc.M205867200

[50] Sreerama N, Woody RW (2004) Computation and Analysis of Protein Circular Dichroism Spectra. Methods in Enzymology 383: 318–351. 15063656 10.1016/S0076-6879(04)83013-1

[51] Nägler DK, Storer AC, Portaro FC, Carmona E, Juliano L, et al (1997) Major increase in endopeptidase activity of human cathepsin B upon removal of occluding loop contacts. Biochemistry 36: 12608–12615. 9376367 10.1021/bi971264+

[52] Barrett AJ (1980) Fluorimetric assays for cathepsin B and cathepsin H with methylcoumarylamide substrates. Biochem J 187: 909–912. 6897924 10.1042/bj1870909 PMC1162479

[53] Nunes GLC, Simões A, Dyszy FH, Shida CS, Juliano MA, et al (2011) Mechanism of Heparin Acceleration of Tissue Inhibitor of Metalloproteases-1 (TIMP-1) Degradation by the Human Neutrophil Elastase. PLoS One 6: e21525. 21731773 10.1371/journal.pone.0021525 PMC3121799

[54] Schneck JL, Villa JP, McDevitt P, McQueney MS, Thrall SH, et al (2008) Chemical Mechanism of a Cysteine Protease, Cathepsin C, As Revealed by Integration of both Steady-State and Pre-Steady-State Solvent Kinetic Isotope Effects. Biochemistry 47: 8697–8710. 18656960 10.1021/bi8007627

[55] Stein RL, Strimpler AM, Hori H, Powers JC (1987) Catalysis by human leukocyte elastase. Aminolysis of acyl-enzymes by amino acid amides and peptides. Biochemistry 26: 2238–2242. 3650109 10.1021/bi00382a025

[56] Xu H, Bush LA, Pineda AO, Caccia S, Di Cera E (2005) Thrombomodulin Changes the Molecular Surface of Interaction and the Rate of Complex Formation between Thrombin and Protein C. J Biol Chem. 280: 7956–7961. 10.1074/jbc.M412869200 15582990

[57] van Holde KE (2002) A hypothesis concerning diffusion-limited protein-ligand interactions. Biophys Chem 101–102: 249–254. 10.1016/s0301-4622(02)00176-x 12488005

[58] Hussain S, Khan A, Gul S, Resmini M, Verma CS, et al (2011) Identification of interactions involved in the generation of nucleophilic reactivity and of catalytic competence in the catalytic site Cys/His ion pair of papain. Biochemistry 50: 10732–10742. 22044167 10.1021/bi201207z

[59] Hussain S, Pinitglang S, Bailey TS, Reid JD, Noble MA, et al (2003) Variation in the pH-dependent pre-steady-state and steady-state kinetic characteristics of cysteine-proteinase mechanism: evidence for electrostatic modulation of catalytic-site function by the neighbouring carboxylate anion. Biochem J 372: 735–746. 12643810 10.1042/BJ20030177 PMC1223443

[60] Gesteira TF, Coulson-Thomas VJ, Taunay-Rodrigues A, Oliveira V, Thacker BE, et al (2011) Inhibitory peptides of the sulfotransferase domain of the heparan sulfate enzyme, N-deacetylase-N-sulfotransferase-1. J Biol Chem 286: 5338–4536. 20129923 10.1074/jbc.M110.100719 PMC3037646

[61] Koti ASR, Krishna MMG, Periasamy N (2001) Time-resolved area-normalized emission spectroscopy (TRANES): a novel method for confirming emission from two excited states, J Phys Chem A. 105: 1767–1771.

[62] Carmona E, Dufour E, Plouffe C, Takebe S, Mason P, et al (1996) Potency and selectivity of the cathepsin L propeptide as an inhibitor of cysteine proteases. Biochemistry 35: 8149–8157. 8679567 10.1021/bi952736s

[63] Coulombe R, Grochulski P, Sivaraman J, Ménard R, Mort JS, et al (1996) Structure of human procathepsin L reveals the molecular basis of inhibition by the prosegment. EMBO J 15: 5492–5503. 8896443 PMC452294

[64] Mason RW, Gal S, Gottesman MM (1987) The identification of the major excreted protein (MEP) from a transformed mouse fibroblast cell line as a catalytically active precursor form of cathepsin L. Biochem J. 248: 449–454. 10.1042/bj2480449 PMC1148562 3435459

[65] Pungercar JR, Caglic D, Sajid M, Dolinar M, Vasiljeva O, et al (2009) Autocatalytic processing of procathepsin B is triggered by proenzyme activity. FEBS J 276: 660–668. 19143833 10.1111/j.1742-4658.2008.06815.x PMC4551429

[66] Fairhead M, Kelly SM, van der Walle CF (2008) A heparin binding motif on the pro-domain of human procathepsin L mediates zymogen destabilization and activation. Biochem Biophys Res Commun 366: 862–867. 18086562 10.1016/j.bbrc.2007.12.062

[67] Horn M, Jílková A, Vondrášek J, Marešová L, Caffrey CR, et al (2011) Mapping the Pro-Peptide of the *Schistosoma mansoni* Cathepsin B1 Drug Target: Modulation of Inhibition by Heparin and Design of Mimetic Inhibitors. ACS Chem Biol 6: 609–617. 21375333 10.1021/cb100411v

[68] Caglic D, Pungercar JR, Pejler G, Turk V, Turk B (2007) Glycosaminoglycans Facilitate Procathepsin B Activation through Disruption of Propeptide-Mature Enzyme Interactions. J Biol Chem 282: 33076–33085. 17726009 10.1074/jbc.M705761200

[69] Novinec M, Grass RN, Stark WJ, Turk V, Baici A, et al (2007) Interaction between human cathepsins K, L and S and elastins: mechanism of elastinolysis and inhibition by macromolecular inhibitors. J Biol Chem 282: 7893–7902. 17227755 10.1074/jbc.M610107200

[70] Roughley PJ, Barrett AJ (1977) The degradation of cartilage proteoglycans by tissue proteinases. Proteoglycan structure and its susceptibility to proteolysis. Biochem J 167: 629–637. 603625 10.1042/bj1670629 PMC1183709

[71] Schenker P, Baici A (2010) Paradoxical interactions between modifiers and elastase-2. FEBS J 277: 2486–2495. 20553487 10.1111/j.1742-4658.2010.07663.x

